# Beyond ‘significance’: principles and practice of the Analysis of Credibility

**DOI:** 10.1098/rsos.171047

**Published:** 2018-01-17

**Authors:** Robert A. J. Matthews

**Affiliations:** Department of Mathematics, Aston University, Birmingham B4 7ET, UK

**Keywords:** statistical inference, significance testing, credibility, replication crisis, Bayesian methods

## Abstract

The inferential inadequacies of statistical significance testing are now widely recognized. There is, however, no consensus on how to move research into a ‘post *p* < 0.05’ era. We present a potential route forward via the Analysis of Credibility, a novel methodology that allows researchers to go beyond the simplistic dichotomy of significance testing and extract more insight from new findings. Using standard summary statistics, AnCred assesses the credibility of significant and non-significant findings on the basis of their evidential weight, and in the context of existing knowledge. The outcome is expressed in quantitative terms of direct relevance to the substantive research question, providing greater protection against misinterpretation. Worked examples are given to illustrate how AnCred extracts additional insight from the outcome of typical research study designs. Its ability to cast light on the use of *p*-values, the interpretation of non-significant findings and the so-called ‘replication crisis’ is also discussed.

## 1. Introduction

Statistical inference plays a key role in the scientific enterprise by providing techniques for turning data into insight. It is therefore striking that the most commonly used technique—statistical significance testing—has prompted grave concern among statisticians almost since its inception [1–3]. Prominent coverage of the so-called replication crisis [4–5] has catalysed widespread debate about the use of significance testing in general, and *p*-values in particular. In March 2016, the American Statistical Association (ASA) issued an unprecedented Statement expressing concern that *p*-values are ‘commonly misused and misinterpreted’ and calling for researchers to ‘steer research into a ‘post *p* < 0.05’ era’ [6]. However, the ASA Statement gives no explicit guidance on how this should be accomplished, stating only that some statisticians ‘supplement or even replace *p*-value’ using methods such as estimation via confidence intervals (CIs), Bayesian methods and false discovery rates. This lack of specific guidance reflects long-standing debate among statisticians about the relative merits of different inferential methods. Yet as various commentators have noted (e.g. [7]), without such guidance research workers can hardly be expected to abandon familiar methods whose output, however unreliable, is regarded as necessary for publication in peer-reviewed journals.

There is unlikely ever to be agreement on a single inferential technique to replace significance testing, not least because of the multi-faceted nature of inference. Nevertheless, both the ASA Statement and its associated Commentaries point to a consensus on the desirable features of any acceptable alternatives:

— They should move the assessment of research findings beyond simplistic ‘pass/fail’ dichotomization. Significance testing notoriously focuses on the *p*-value threshold of 0.05, below which findings are deemed ‘significant’ and worth further study, and above which they are rejected as both ‘non-significant’ and (often unjustifiably) evidence of no effect.
— They should allow individual study findings to be put into the context of existing knowledge, allowing their intrinsic plausibility to be assessed in transparent and quantitative terms. Significance testing offers no such mechanism beyond arbitrary shifting of the *p* = 0.05 threshold in an attempt to reflect greater or lesser degrees of scepticism.
— Their output should have a clear and intuitive interpretation of direct relevance to the substantive (i.e. non-null) hypothesis, in contrast to *p*-values, whose definition is notoriously convoluted and of only indirect relevance even to the null hypothesis.
— Ideally, their application should require only conventional summaries of evidence familiar to non-specialists, while still allowing the use of more sophisticated inferential methods without substantial additional information.

In what follows, we introduce a novel methodology designed to meet these requirements: the Analysis of Credibility (AnCred). Its origins lie in a technique originally developed for assessing statistically significant outcomes of clinical trials [8,9] which has since found application in healthcare evaluation [10], epidemiology [11], health risk assessment [12] and interpretive issues in inference [13]. The technique leads to a simple metric, the *Critical Prior Interval* (CPI) that allows new findings to be set in the context of existing knowledge and insight.

This paper presents the generalization of this technique into a methodology capable of assessing claims of both statistical significance *and* non-significance in both the presence and absence of existing insight. The result is an inferential toolkit which can be used alongside standard statistical significance testing, extracting extra insight from findings expressed using conventional summary statistics.

## 2. Moving beyond the *p*-value dichotomy

The principal concern about conventional significance testing is its promotion of simplistic assessments of new findings (see [14–16]). This is most starkly demonstrated by the *p*-value threshold, widely adopted following publication of R. A. Fisher's classic *Statistical Methods for Research Workers* [17], according to which results are either statistically significant (*p* ≤ 0.05) or non-significant (*p* > 0.05). By encouraging practices such as ‘data dredging’, this pass/fail dichotomy has been identified as leading to ‘considerable distortion of the scientific process' [6].

Any methodology capable of moving beyond this dichotomy must be capable of extracting more insight from study data. This, in turn, means adopting a more informative metric than *p*-values, which have no simple relationship to effect size or weight of evidence, and offer no simple means of being combined with other sources of insight. These deficiencies have led to the increasing use of confidence intervals (CIs), now widely recognized as concise but more informative summary statistics (e.g. [18,19]). Expressed in terms of lower and upper bounds (*L*, *U*), a 95% CI can be ‘unpacked’ to give a central estimate of the effect size and a measure of evidential weight. That, in turn, allows the CI from a specific study to be combined with CIs representing other sources of insight, allowing the results to be set in context. The means to do this lie within the framework of Bayesian inference (e.g. [20]), the use of which bring the added benefit of allowing CIs to be interpreted as so-called *credible intervals.* Unlike CIs, these represent the range of values within which the effect size lies with the stated probability (e.g. 95%); despite widespread misconceptions [21] conventional CIs can only be interpreted in this way on the assumption of a complete absence of pre-existing insight. Given the accumulation of quantitative insight across many disciplines, such an assumption is rarely justifiable.

This combination of CIs and Bayesian methods forms the mathematical framework of AnCred. However, to achieve its goal of extracting greater insight from findings, this framework must be turned into an inferential process. To this end, we introduce the novel concept of subjecting claims of significance and non-significance to *fair-minded challenge*.

## 3. Inference based on fair-minded challenge

Bayesian methods provide the mathematical framework for combining existing (prior) insight with new findings to arrive at an updated (posterior) level of insight. Symbolically, the process can be represented as

3.1 Prior insight+Data likelihood→Posterior insight.

Conventionally, the process runs from left to right: a prior probability distribution representing existing knowledge is combined using Bayes's Theorem with the so-called likelihood capturing the evidential weight provided by the new data. If the 95% credible interval of the resulting posterior distribution then excludes no effect, the findings are said to be credible at the 95% level. This process is open to various well-known objections: the prior distribution could be based on misguided subjective opinion, for example, or be hand-picked to ensure that the resulting posterior achieves credibility. This ‘Problem of Priors’ has generated a substantial literature dating back centuries (see [22]). However, as Good pointed out nearly 70 years ago [23], it is entirely legitimate to run (3.1) from right to left, and thus *deduce* the prior needed to produce a posterior distribution implying the findings are credible. Having been extracted from the (objective) findings of the study, the standard criticisms of the choice of prior then no longer arise.

This inversion of Bayes's Theorem provides the mathematical framework by which AnCred goes beyond the dichotomous process of significance testing. It does this by challenging the claim that a finding is significant or non-significant as follows:

1. The summary statistic for the findings is used to deduce the range of prior effect sizes—the CPI—capable of challenging the credibility of the claim of significance/non-significance, in the sense of leading to a posterior interval that includes/excludes no effect.
2. Comparison of this CPI with effect sizes supported by prior evidence then allows the credibility of the claim to be assessed in transparent and quantitative terms.

As is shown in the appendix, the inversion of Bayes's Theorem requires the form and location of the prior distribution to be specified. This in turn depends on whether the claim being challenged is of significance or non-significance. In both cases, the necessary characteristics of the prior distribution can be established by invoking the *Principle of Fair-Minded Challenge.*

In the case of statistically significant results, this implies challenge on the basis of fair-minded *scepticism*. That is, the claim of statistical significance is challenged by a prior modelling the belief of a hypothetical sceptic who regards the absence of an effect to be the most likely reality. Clearly, even the most compelling evidence can be dismissed by adopting a suitably restrictive prior. Such gaming of the inferential process is countered via the concept of *fair-mindedness*, by which the sceptic accepts that while believing no effect to be the most likely reality, there remains a finite probability of an effect of some magnitude.

Similarly, non-significant findings are challenged on the basis of fair-minded *advocacy.* This requires a prior modelling belief in the existence of an effect of some non-zero magnitude, while accepting that this magnitude cannot exceed some reasonable bound.

As we now show, in both cases this process of fair-minded challenge moves the assessment of findings beyond the usual simplistic pass/fail dichotomy to focus instead on the significance or otherwise of a finding in the context of current knowledge and insight.

## 4. AnCred for statistically significant findings

Following [9], we model the stance of fair-minded scepticism using a Normal distribution centred on no effect, whose 95% tails are set by the requirement that when combined with the likelihood from the data, the resulting posterior distribution renders the finding no longer credible at the 95% level. The range of prior values capable of achieving this constitutes the CPI; only if existing knowledge supports values lying *outside* the CPI can the claim of statistical significance be deemed to be credible at the 95% level.

The appendix derives the CPI for findings from comparative studies (e.g. intervention versus control groups) expressed in the two most widely encountered formats: differences between means or proportions, and ratios. In the case of a statistically significant difference stated as a 95% CI of (*L*, *U*), the CPI has lower and upper bounds (−SL, +SL) where SL is the *Scepticism Limit* calculated from the data via (see appendix §A.2 below, or [24])

4.1 $$\text{SL}=\frac{{\left(U-L\right)}^{2}}{4\sqrt{UL}}.$$

Only if prior evidence exists for differences lying *outside* the range (−SL, +SL) can the claim of significance be deemed credible at the 95% level. In the case of statistically significant *ratios* the CPI has bounds of (1/SL, SL) where

4.2 $$\text{SL}=\text{exp}\left[\frac{\text{l}{\text{n}}^{2\;}(U\text{/}L)}{4\sqrt{\text{ln( }U\text{) ln( }L\text{) }}}\right].$$

**Figure 1.**
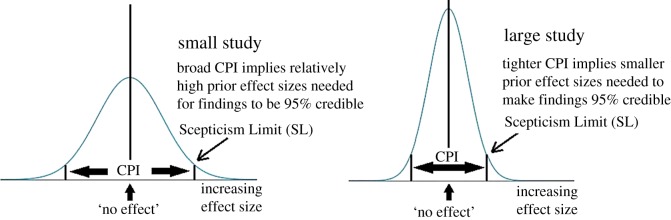
The sceptical CPI used to challenge claims of statistical significance. Large studies have relatively low SLs, making sceptical challenge harder to sustain using existing evidence.

Note that the CPI in both expressions depends on *both* the lower and upper bounds of the CI summarizing the data. This reflects the fact that the credibility of a finding depends critically on evidential weight, as indicated by the width of the interval (*L*, *U*). Findings from small studies with broad CIs will have broad CPIs. As these encompass relatively large effect sizes, they offer considerable latitude for successful challenge by a fair-minded sceptic using existing insight. By contrast, findings from large studies will typically have tighter CPIs, reflecting their greater evidential weight. This in turn narrows the range of effect sizes available to the sceptic for a successful challenge (figure 1).

The concept of fair-minded challenge is both intuitive and makes use of all the evidential content in the CI summarizing the finding. It also requires that a claim of statistical significance be set in the context of existing knowledge. This can come from, for example, previous studies of the same research question, studies of broadly similarly phenomena, elicitation from experts, or combinations of these and other sources of insight. AnCred allows all such sources to be used to substantiate a claim of credible significance. However, it also requires their explicit specification in quantitative terms, in order to demonstrate the existence of effect sizes outside the Scepticism Limit (SL). This feature of AnCred thus makes an important extra demand of conventional significance testing, which focuses solely on whether a summary CI excludes values corresponding to no effect, while taking no quantitative account of evidential weight or existing knowledge.

## 5. AnCred for non-significant findings

Under AnCred, claims of non-significance are challenged on the basis of *fair-minded advocacy*, modelled by a prior distribution that depends on the nature of the substantive hypothesis. For simplicity, we will focus on cases where the substantive hypothesis implies differences in means exceed zero, and ratios exceed unity (e.g. better test scores, or higher odds ratios for mortality); the results where these inequalities are reversed follow by symmetry.

**Figure 2.**
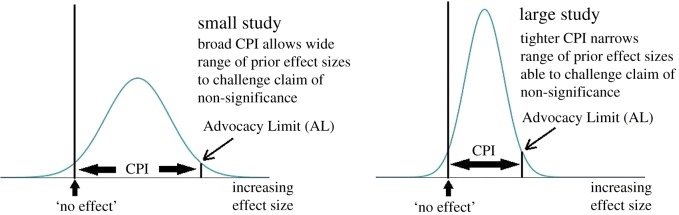
The advocacy CPI for assessing claims of statistical non-significance. Non-significant findings from large studies have relatively low ALs, limiting the range of effect sizes available to advocates to challenge the claim of non-significance.

In these cases, the advocacy distribution has a lower bound set by the absence of an effect; this reflects the advocate's view that there is most likely *some* positive effect. The upper bound is set by the advocate's acceptance that the magnitude of any positive effect must be bounded (figure 2). In the case of differences between means and proportions, these two conditions imply that claims of non-significance can be challenged by advocates of a (positive) effect if they are able to cite prior evidence of effect sizes lying within the advocacy CPI of (0, AL) where AL is the *Advocacy Limit.* For differences between means and proportions stated as a CI of (*L*, *U*) the value of AL is (see appendix §A.3)

5.1 $$\text{AL}=\frac{-(U+L)}{2UL}{\left(U-L\right)}^{2},$$

while for findings expressed as ratios, the CPI is (1, AL) where

5.2 $$\text{AL}=\text{exp}\left[-\frac{\text{ln( }UL\text{) l}{\text{n}}^{2\;}(U\text{/}L)}{2\text{ln( }U\text{) ln( }L\text{) }}\right]\;.$$

As with the sceptical prior, the presence of both *U* and *L* in (5.1) and (5.2) shows that AnCred reflects the intuition that the credibility of *non*-significant findings depends on their evidential weight as reflected by the width of their 95% CI. Studies with weak levels of non-significance will lead to broad CPIs with considerable latitude for successful challenge using existing knowledge. By contrast, strongly non-significant findings will lead to more restrictive ALs and narrower CPIs, as only these have enough evidential weight to produce posterior distributions that exclude the null. AnCred thus highlights the dangers of regarding non-significance as a sharp cut-off, beyond which a finding can be summarily dismissed as ‘negative’. Rather, non-significance is, like significance, a matter of *degree*: some findings are more compellingly non-significant than others. The stronger (weaker) the negative evidence, the harder (easier) it becomes for advocates to challenge claims of non-significance (figure 2).

The criterion for determining the credibility of a claim of non*-*significance follows from the requirement that advocates of the reality of an effect are not able to cite values within the advocacy CPI capable of challenging the claim. As the advocacy CPI is necessarily asymmetric about no effect, the criterion depends on the nature of the substantive hypothesis, but remains simple and intuitive: the non-significance of a finding is credible if the central estimate M lies on the opposite side of the null line from effect sizes consistent with the substantive hypothesis. Thus, for differences of means or proportions, if the substantive hypothesis is for effect sizes greater (less) than zero, a non-significant finding with *M* less (greater) than zero is also credible. The criterion for ratios follows by replacing zero with unity.

It should be emphasized that advocates of the existence of an effect can still challenge the non-significance, but not within the definition of fair-minded advocacy defined by AnCred. Instead, they must invoke custom-made priors to model their beliefs, and make the case for their choice on the basis of prior knowledge and insight.

## 6. Unprecedented findings and intrinsic credibility

Thus far, we have assumed there exists quantitative prior evidence suitable for comparison with the Scepticism and Advocacy Limits (ALs) generated by AnCred. This is not always the case, however; by its very nature, scientific research can lead to findings without obvious precedent, and for which relevant prior evidence does not exist. Such ‘out of the blue’ findings commonly emerge from exploratory studies, and are perhaps most familiar in epidemiology, which is replete with claims of seemingly implausible causal connections between some environmental exposure and negative health effects (see [25,26]).

As noted earlier, the assessment of evidence in the absence of relevant prior insight is a long-standing challenge to inference in general, and Bayesian methods in particular, where it constitutes the notorious ‘Problem of Priors’. AnCred provides a framework for handling these problematic cases via the concept of *intrinsic credibility*.

**Figure 3.**
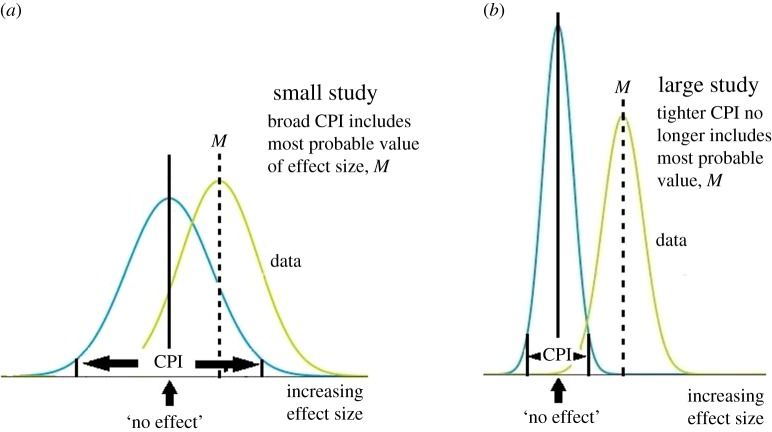
Small studies (*a*) have CPIs so broad they may encompass the most probable effect size, *M*. Their statistical significance will then lack intrinsic credibility. In contrast, large studies (*b*) have relatively narrow CPIs less likely to encompass *M*, and thus more likely to make their statistical significance intrinsically credible.

Recall that AnCred allows the credibility of claims of statistical significance to be challenged if existing evidence points to effect sizes lying within the CPI of the findings. In the case of ‘out of the blue’ findings, however, the only existing insight is from the study itself. The most probable magnitude of the effect size is then given by the mode of the data-driven likelihood, which for normally distributed parameters is the central estimate *M*. Findings with relatively low evidential weight will, however, have broad CPIs, and these may be so large they include the central estimate *M*. This implies that the statistical significance of the finding can only be deemed credible if there is already prior evidence for effect sizes greater than that being claimed. This is clearly problematic for unprecedented ‘out of the blue’ findings, for which there is no evidence other than that provided by the study itself. In such cases, the finding may be said to be statistically significant but lacking *intrinsic credibility*. On the other hand, for unprecedented findings whose central estimate lies outside the CPI, we can say that the claim of statistical significance is *intrinsically* credible. Put colloquially, the unprecedented study has made its case ‘in its own terms’, without needing pre-existing insight. This is clearly a more onerous demand for smaller studies: their CPIs are broader, and thus require larger effect sizes to achieve intrinsic credibility (figure 3).

The criterion for a claim of statistical significance for an unprecedented finding being intrinsically credible is that *M* lies outside the sceptical CPI, which for differences in means and proportions implies that (see appendix §A.4)

6.1 M<αL, 

where $\;\alpha =1+\varphi +\sqrt{1+2\varphi }∼4.6757$ and $\varphi =(1+\sqrt{5})/2∼1.618\ldots$

For ratios the corresponding requirement is

6.2 $$M<\;L^{4.68}.$$

Remarkably, these inequalities can both be re-cast as a *p*-value threshold, below which a claim of statistical significance may be said to be intrinsically credible. At the conventional 95% level the criterion for intrinsic credibility is equivalent to a *p*-value threshold of

6.3 p≤0.013.

While convenient and familiar, it must be stressed that this *p*-value threshold serves an entirely different purpose from those used in conventional significance testing. Through their (mis-) interpretation as a time-invariant measure of the inferential value of findings, *p*-value thresholds are used to justify pass/fail categorizations which hold for all time, regardless of the emergence of new evidence. Under AnCred, by contrast, (6.3) determines whether an unprecedented but statistically significant finding requires support from external sources of evidence if it is also to achieve credibility at the 95% level. Flagging this up is clearly valuable in assessing unprecedented studies where prior evidence is lacking. Equally clearly, however, failure to meet the threshold for intrinsic credibility cannot be grounds for dismissing a finding, as the necessary supporting evidence may emerge over time. As such, attempts to interpret (6.3) in terms of conventional ‘significance’, evidential weight, Bayes Factor bounds etc. are inappropriate.

The criterion for determining if the statistical *non-­*significance of an unprecedented finding is also intrinsically credible follows from the discussion at the end of the previous section.

## 7. Illustrative examples of the use of AnCred

Having described its fundamental concepts and their implications for conventional significance testing, we now show how to apply AnCred to prototypical claims of statistical significance and non-significance.

To demonstrate the use of AnCred we follow [27] and consider two sets of studies of the same research question, in the form of five small (*N* = 100) and five large (*N* = 1000) randomized trials (table 1). Each trial compares the response rates of the *N*/2 subjects in the intervention arm to those in the control arm. There is a different response rate in each intervention arm, while that in the control arm is fixed at 20%, and the substantive hypothesis is for odds ratios (ORs) > 1. For both sets, the first two trials have statistically significant differences; the next two have non-significant differences with central estimates consistent with positive effects (i.e. ORs > 1), while the final variant has a central estimate consistent with a negative effect (OR < 1). The table gives the resulting standard summary statistics, *p*-values and 95% CI bounds, while the last two columns gives the outcome of applying AnCred. The first of these gives the SL or AL capable of challenging the claim on the basis of existing knowledge and insight. These are calculated from the 95% CI for the trial results using (4.2) and (5.2) above. The second shows whether the claim of significance or non-significance for an unprecedented finding lacking prior support meets the requirement for intrinsic credibility.

Conventionally, the assessment of such findings focuses on the central estimate of the 95% CI and especially on whether its bounds exclude values implying no effect (or, equivalently, whether *p* < 0.05). By those criteria, the most impressive finding in table 1 appears to be S1, with its large central estimate OR of 4.33 and high level of significance (*p* = 0.001). Conventional analysis of the much larger trial L1, meanwhile, would highlight its more modest central estimate. Nevertheless most researchers would regard the outcome of L1 as in some sense more ‘convincing’, on the grounds that it is based on a much larger trial. This intuition cannot be justified by appeal to the *p*-values, however, as they are the same for both studies. The 95% CIs do give more justification for preferring L1 over S1, as the former is tighter than the latter, reflecting its greater evidential weight.

**Table 1.** Prototypical examples of AnCred assessments of the credibility of statistically significant and non-significant findings in both the presence and absence of prior evidence. OR, odds ratio.

						AnCred	
trial variant	intervention response rate	central estimate (OR)	*p*-value	95% CI lower bound (OR)	95% CI upper bound (OR)	scepticism/ advocacy limit (OR)	intrinsically credible if no prior evidence?
	**small trials (*n* = 100)**						
	statistically significant results						
S1	52%	4.33	0.001	1.78	10.5	SL = 2.0	yes
S2	40%	2.67	0.03	1.09	6.52	SL = 7.3	no
	statistically non-significant results						
S3	36%	2.25	0.08	0.91	5.55	AL ≥ 100	no
S4	26%	1.41	0.48	0.55	3.59	AL = 4.8	no
S5	18%	0.88	0.80	0.32	2.39	none available	yes
**large trials (*n* = 1000)**							
	statistically significant results						
L1	29%	1.63	0.001	1.22	2.18	SL = 1.24	yes
L2	26%	1.41	0.025	1.045	1.89	SL = 1.69	no
	statistically non-significant results						
L3	25%	1.33	0.06	0.99	1.80	AL ≫ 100	no
L4	23%	1.20	0.25	0.88	1.62	AL = 2.9	no
L5	19%	0.95	0.74	0.67	1.33	none available	yes

AnCred replaces this kind of fuzzy, qualitative judgement with a principled and well-founded quantitative assessment. First, the intuition that L1 is more compelling than S1 is confirmed by comparison of their SLs. Trial S1 requires prior evidence of effect sizes exceeding an OR of 2.0 if its claim of statistical significance is to be credible at the 95% level. By contrast, L1 requires prior evidence for ORs above 1.24, a substantially less demanding value resulting directly from its greater evidential weight. Second, AnCred allows assessment of whether S1 and/or L1 actually are credibly significant at the 95% level to be performed transparently and quantitatively by comparing their SLs with effect sizes based on extant evidence.

For trials S2 and L2, their *p*-values of approximately 0.03 show that both are again formally statistically significant. As before, however, AnCred confirms the intuition that the larger trial L2 makes a stronger case than S2, despite the similar *p*-values: while the latter requires prior support for effect sizes exceeding an OR of 7.3, L2 can achieve credible significance from the much less demanding SL OR of 1.69. These two trials also highlight the ability of AnCred to add value to standard significance testing in the case of unprecedented findings. If S2 and L2 were both exploratory trials, both would be deemed to have found statistically significant evidence of an ‘out of the blue’ effect. However, AnCred shows that neither claim is credible as they both fail to meet the criterion for intrinsic credibility of *p* < 0.013. It is notable that despite being 10 times larger than S2, the statistical significance of L2 still lacks intrinsic credibility. It must await the emergence of evidence for effect sizes exceeding ORs of 1.69 before its statistical significance can be deemed credible.

The implications of AnCred are also striking when applied to the outcome of trials S3 and L3, whose *p*-values are in the range 0.05 < *p* < 0.10, leading to euphemistic descriptions such as ‘fairly significant’, ‘approaching significance’ etc. [28]. Despite both being formally non-significant, the 95% CIs of S3 and L3 lead to very high ALs. Thus neither trial puts useful constraint on advocates of the substantive hypothesis who seek to challenge the claim of non-significance. This reflects the fact that while the 95% CIs of both trials encompass no effect, their central estimates *M* both exceed unity. As such, the bulk of their evidential weight remains consistent with the substantive hypothesis of ORs > 1. AnCred thus confirms the widely held belief that findings lying in the inferential twilight zone of 0.05 < *p* < 0.10 are inferentially unsatisfactory. Simply put, both S3 and L3 lack the evidential weight to make a credible claim for or against statistical significance, and only more data can resolve the ambiguity.

By contrast, trials S4 and L4 show how AnCred can extract considerable insight from *non*-significant findings, so often automatically dismissed as ‘negative’. The moderately high *p*-values of both S4 and L4 (0.48 and 0.25, respectively) show that both are formally non-significant. However, as both also have central estimates exceeding ORs of 1.0, it is clear that both still provide *some* support for the substantive hypothesis. AnCred reflects this via ALs for these two studies of 4.8 and 2.9, respectively. Thus while the studies are non-significant, the credibility of this claim can still be challenged by advocates who accept effect sizes are unlikely to exceed ORs of 4.8 and 2.9, respectively. AnCred thus shows how the weight of (negative) evidence in studies constrains the effect size that advocates can still defend. As with the sceptical challenge of statistically significant results, advocates wishing to resist this constraint can do so via appeal to prior evidence. As one would expect—and as we saw with S3 and L3—studies with modest evidential weight against an effect—and thus high ALs—are more easily challenged by advocates than large studies with tight ALs.

Finally, trials S5 and L5 illustrate the case of compelling evidence against the existence of an effect. Their central values both lead to ALs lying outside the range used in AnCred to model fair-minded advocacy. They therefore offer no effect sizes to advocates of ORs > 1 capable of challenging the claim of non-significance, which is therefore credible under AnCred. This is a direct consequence of both having the bulk of their evidential weight at ORs < 1, contradicting the substantive hypothesis.

As noted earlier, advocates of the existence of an effect can of course still challenge the claim of non-significance, but to do so they must invoke custom-made priors to model their beliefs, justifying their choice on the basis of prior knowledge and insight.

## 8. AnCred and ‘discordant’ findings

One of the principal drivers of the ASA's Statement was concern over the failure rate of attempted replications of highly cited findings [6]. Such failures are usually judged on the basis of significance testing, with discordant findings being those lying on opposing sides of the *p* = 0.05 threshold. AnCred shows this to be an unreliable dichotomy.

Suppose trial S1 in table 1 was an exploratory study to investigate a substantive hypothesis such that ORs > 1. Conventionally, the central OR of 4.3 combined with its high statistical significance (*p* = 0.001) would be deemed impressive evidence of efficacy. AnCred adds further credence to the finding, by showing that despite being exploratory and thus lacking prior evidential support, it possesses sufficient evidential weight for its statistical significance to be intrinsically credible. Suppose now that an attempt to replicate this claim was made in a second, much larger trial such as L3 in table 1. Attention would typically focus on the fact that L3 has a 95% CI encompassing OR = 1 (or equivalently that the trial's *p*-value exceeds 0.05), implying the finding is not significant. This in turn would lead to L3 being classified as a ‘negative’ finding, and thus to S1 and L3 being ‘discordant’. Furthermore, given the size of L3, its apparent failure to confirm S1 would be regarded as strong evidence against the substantive hypothesis.

The unreliability of this reasoning is immediately apparent from the summary statistics of S1 and L3, which shows that *both* trials have central estimates exceeding unity. Thus despite the small size of S1 and the non-significance of L3, in both cases the bulk of their evidential weight is consistent with the substantive hypothesis.

This highlights the fallacy of confusing absence of evidence with evidence of absence [29,30]. AnCred provides additional protection against this fallacy by taking explicit account of the full evidential content of the summary statistics. First, it shows that despite being relatively small and lacking prior evidential support, the effect size found in S1 is both statistically significant and intrinsically credible. Second, it shows that despite its size, L3's claim of non-significance lacks credibility at the 95% level for all reasonable ALs. This is essentially because the central value of L3 is such that the bulk of its evidence is still consistent with the existence of an effect.

AnCred thus shows that S1 and L3 are *not* discordant in the sense of providing impressive evidence for opposing effects; indeed, the central—and thus most probable—effect size estimates are consistent with each other, both pointing to the reality of a positive effect. This is supported by a simple ‘meta-analysis’ of the two studies: combining the relevant numbers in the various arms of S1 and L3 leads to an updated OR of 1.51, with a 95% CI of (1.14, 2.00), which remains highly statistically significant (0.004). It is also credible at the 95% level for ORs exceeding 1.3, which is a somewhat tighter CPI than S1 alone. Thus, rather than diluting the evidence for an effect, the addition of the supposedly ‘discordant’ larger study has in fact pushed sceptics into a somewhat tighter corner. Indeed, an advocate of the existence of an effect could challenge L3's non-significance simply by pointing out that the entire 95% CI of the original study S1 lies comfortably within L3's AL.

The outcome of subjecting these two studies to AnCred is thus in stark contrast to the standard assessment of discordancy, with its misguided focus on the location of tails and neglect of central values.

## 9. Conclusion

After decades of debate, there is now a consensus among statisticians that misuse and misinterpretation of significance testing is routine, widespread and threatens the credibility of both the statistical community and the scientific enterprise. In the words of Berry: ‘Patients with serious diseases have been harmed. Researchers have chased wild geese… The effects extend to the public and affect the lay person's understanding and appreciation of science’ [31]. Nevertheless, there remains no consensus on the way forward, despite a plethora of suggestions ranging from a simple tightening of the *p*-value threshold [32] through false discovery risk methods [33,34] and Bayes Factors [35] to sophisticated Bayesian hierarchical modelling [36].

As a response to the call to move towards the ‘post *p* < 0.05 era’, the AnCred has been developed in the spirit of evolution rather than revolution. Rather than requiring the wholesale replacement of familiar methods and metrics, AnCred provides a framework for extracting more insight from them, while reducing the risk of inferential misinterpretation. Its focus on CIs leads to more informative summaries of findings than simple statements of *p*-values; these also allow more sophisticated techniques to be applied without requiring substantial additional information. By incorporating them into a Bayesian framework, AnCred also gives CIs a central role in the accumulation of insight of direct relevance to the substantive hypothesis, again in marked contrast to *p*-values.

The application of AnCred allows findings to be categorized using the familiar labels of statistical significance and non-significance, but crucially it does not end there. Instead, it subjects each claim to *fair-minded challenge*, on the basis of scepticism of the substantive hypothesis in the case of statistically significant results, and advocacy of the existence of an effect for non-significant results. Thus the traditional simplistic dichotomization centred on *p*-values is replaced by a process that encourages transparent discussion of findings in the context of existing knowledge. A finding may be statistically significant, yet possess so little evidential weight that it lacks credibility according to current extant evidence. However, this may change in the light of subsequent research; AnCred allows reconsideration of findings in a way precluded by the one-shot, pass/fail dichotomization of conventional significance testing. At the same time, the use of SLs and ALs compels researchers to be transparent and explicit in their use of extant evidence to defend or challenge a specific finding.

Cutting-edge research necessarily produces findings lacking any obvious precedent, and assessing such findings has been a long-standing inferential challenge. AnCred offers an approach based on the concept of *intrinsic* credibility, by which claims of significance/non-significance can be assessed even in the absence of external prior evidence.

As with any inferential method, AnCred rests on assumptions and models that are open to challenge. Notably, it requires findings to be stated in terms of CIs, assumes (log)normality and models the concepts of fair-minded scepticism and advocacy in ways that may not be appropriate in specific circumstances. The basic AnCred framework can, however, be modified to incorporate more complex models, albeit at the cost of ease of use and interpretation. Abandoning the use of conjugate distributions would, for example, necessitate the use of computational methods beyond the capabilities of most research workers, for whom AnCred has principally been devised. In addition, while the focus of this paper has been on assessing comparative studies, there is clearly substantial scope for extending the same approach to other aspects of inference, such as regression and correlation analysis.

In the meantime, AnCred already has the potential to cast new light on implausible, ‘negative’ and ‘discordant’ claims in the existing research literature. Given the proven inadequacies of conventional significance testing, such retrospective analysis is likely to add urgency to calls to move to a ‘post *p* < 0.05 era’.

## Data accessibility

This article has no additional data.

## Appendix

**A.1. General framework**

The purpose of AnCred is to move beyond standard significance testing by assessing findings on the basis of their evidential weight, and their credibility in the context of existing knowledge. It does this by determining the range of effect sizes capable of challenging a claim of significance or non-significance. If effect sizes lying outside this range—known as the CPI—can be justified by the finding itself or by existing insight and knowledge, then the finding can be deemed *credibly* significant/non-significant. The process of challenge is operationalized via Bayes's Theorem which is used to deduce the prior distribution capable of producing a posterior distribution that just encompasses values corresponding to no effect. The study finding itself is modelled using the Normal distribution N(μ,ϕ), leading to the familiar format of a 95% CI of (*L*, *U*) where

A 1 $$L=\mu -1.96\sqrt{\phi }\;\;\;\text{and}\;\;\;U=\mu +1.96\sqrt{\phi }\text{.}$$

The prior distribution $N(\mu_{o},\;\phi_{o})$ capable of mounting the challenge is deduced from the data by inverting Bayes's Theorem, subject to the constraint that the resulting posterior distribution $N(\mu_{P},\;\phi_{P})$ gives a 95% credible interval $\;(L_{P},\;U_{P})\;$that just includes no effect. The means and variances of the likelihood, prior and posterior satisfy the standard equations (see [20])

A 2 $$\frac{1}{\phi_{P}}=\frac{1}{\phi }+\frac{1}{\phi_{o}},\;\frac{\mu_{P}}{\phi_{P}}=\frac{\mu }{\phi }+\frac{\mu_{o}}{\phi_{o}}.$$

The form of the CPI distribution depends on whether the claim being challenged is of statistical significance or non-significance. We address each case in turn. For brevity and simplicity only the CPIs for differences in means and proportions are derived; the corresponding expressions for ratios follow directly from the transformation L→ln( L)  etc. Unless otherwise stated, we also adopt the convention that differences and ratios *exceeding* the null value constitute support for the substantive hypothesis. While we focus on conventional 95% intervals, the credibility level of the CPI can take any value and depends solely on that used to specify *L* and *U*; for example, if the latter are 99% bounds, the credibility level of the CPI will also be 99%.

**A.2. The CPI for statistically significant results**

The parameters of the prior distribution capable of challenging such findings are specified using the Principle of Fair-Minded Challenge (see main text). For statistically significant results, this leads to the *fair-minded scepticism* prior (figure 1, main text). The corresponding CPI is symmetrically centred on $\mu_{o}=0$ corresponding to no effect being deemed the most likely value by the sceptic; then for a substantive hypothesis of differences exceeding zero, the 95% upper bound is given by a *Scepticism Limit* of $\text{SL}=\mu_{o}+1.96\sqrt{\phi_{o}}$ with prior variance $\phi_{o}$. As $\mu_{o}=0\;$ we have

A 3 $$\mathrm{SL}=\;1.96\sqrt{\phi_{o}}.$$

For a finding to be credible at the 95% level, the posterior distribution must satisfy the inequality $\mu_{P}-1.96\sqrt{\phi_{P}}\geq 0$. Thus at the critical value, we have

A 4 $$\mu_{P}=\;1.96\sqrt{\phi_{P}}.$$

The likelihood capturing the study findings is summarized via a 95% CI of (*L*, *U*), from which both μ and φ can be extracted. We now have the means and variances needed to apply (A 2) and solve for the CPI in the form of a 95% CI (−SL, +SL) where

A 5 $$\text{SL}=\frac{{\left(U-L\right)}^{2}}{4\sqrt{UL}}.$$

The corresponding result for ratios follows by applying the transformation U→ ln⁡(U) etc. leading to a CPI of (1/SL, SL), where SL is given by equation (4.2) in the main text.

**A.3. The CPI for statistically non-significant results**

For non-significant results, the Principle of Fair-Minded Challenge implies that the parameters of the prior distribution capable of challenging such findings are given by the *fair-minded advocacy* prior (figure 2, main text) appropriate to the substantive hypothesis under test. For studies where the substantive hypothesis is that differences exceed zero, the *advocacy CPI* has a lower bound at $\mu_{o}=0$ and an upper bound set by the *Advocacy Limit* (AL) which satisfies the conditions

A 6 $$0=\mu_{o}-\;1.96\sqrt{\phi_{o}},\;\mathrm{AL}=\mu_{o}+\;1.96\sqrt{\phi_{o}}.$$

As before, the posterior distribution is required to be such that $0=\mu_{P}-\;1.96\sqrt{\phi_{P}}$ so that $\mu_{P}=1.96\sqrt{\phi_{P}}$, while both μ and ϕ are extracted from the study outcome CI of (*L*, *U*). Inserting the various terms into the Bayesian conditions (A 2) and solving for AL, we find that the CPI for non-significant findings in this case is (0, AL) where

A 7 $$\text{AL}=\;-\frac{(U+L){\left(U-L\right)}^{2}}{2LU}.$$

If extant evidence points to effect sizes lying in the range (0, AL), advocates of the existence of a (positive) effect can challenge the claimed non-significance of the finding. For the criterion necessary for non-significance to be credible, note that as *L* and *U* for non-significant results have opposing signs, the denominator of (A 7) is always negative. Thus AL < 0  ∀ M < 0 which by definition cannot lie within the advocacy CPI. Thus advocates of a (positive) effect cannot invoke any values from existing evidence capable of challenging the non-significance. Hence when the substantive hypothesis is for differences exceeding zero, a claim of non-significance is always credible under AnCred if *M* < 0. The equivalent criterion for ratios follows from the usual transformation M→ln(M), leading to *M* < 1.

**A.4. Intrinsic credibility of statistically significant findings**

Intrinsic credibility determines whether an unprecedented finding possesses sufficient evidential weight to be regarded as credible in its own right. In AnCred this is modelled by requiring that the most probable value of the finding lies outside its own CPI, thus making it credible even in the absence of prior evidence. In the case of statistically significant findings, let the central estimate and CI for the quantity of interest be *M*(*L*, *U*), where *L* > 0. Then in the absence of prior insight, the most probable value for the effect size is the *mode* of the likelihood, which in the case of the Normal distribution is *M*. Thus for a statistically significant result to have intrinsic credibility, *M* must lie outside the CPI that models fair-minded scepticism. In the case of differences in means and proportions, equation (4.1) in the main text implies

A 8 $$M>\;\frac{{\left(U-L\right)}^{2}}{4\sqrt{UL}}\;\text{.}$$

Let *U* = *kL*, *k* > 1. Then *M* = (*k* + 1)L/2, and (A 8) leads to a critical value *k* such that

$$\frac{\left(k+1\right)}{2}=\;\frac{{\left(k-1\right)}^{2}}{4\sqrt{k}}.$$

This leads to a unique relevant root of

$$k=2+\sqrt{5}+2\sqrt{2+\sqrt{5}}∼8.3524\ldots$$

As *M* = (*k* + 1)*L*/2, the criterion needed for a statistically significant result to be intrinsically credible becomes

A 9 $$M<\alpha L\;\text{where}\;\alpha =1+\varphi +\;\sqrt{1+2\varphi }∼4.6762\ldots ,$$

and $\varphi =(1+\sqrt{5})/2∼1.618\ldots \;$ is the so-called Golden Ratio. The appearance of an upper bound reflects the fact that the likelihood representing the data must contain sufficient evidential weight—and thus be sufficiently compact—to ‘make its case’ in its own terms. The equivalent result for the intrinsic credibility of statistically significant ratios follows from the usual logarithmic transformation, leading to

A 10 $$M<L^{4.68}.$$

Remarkably, both (A 9) and (A 10) are equivalent to a specific *p*-value threshold. In the case of (A 9), for findings stated as conventional 100 *k*% CIs of (*L*, *U*), the *p*-value is

A 11 $$p_{K}=2\left\{1-\Phi \left[z_{K}\left(\frac{U+L}{U-L}\right)\right]\right\}\text{, }$$

where Φ[…] is the Normal cumulative function and *z*_0.90_ = 1.645, *z*_0.95_ = 1.960, *z*_0.99_ = 2.576 etc. As *U* = 2*M* − *L* and *M* < 4.6762*L* from (A 9), the threshold in the standard case of differences in means and proportions summarized by a 95% CI is

A 12 $$p_{0.95}<2\left\{1-\Phi \left[1.96\left(\frac{1}{1-1/4.6762}\right)\right]\right\}\;\;=0.0127.$$

The same threshold applies to findings expressed as 95% CI for ratios. Only unprecedented findings with *p*-values meeting this upper bound possess sufficient evidential weight to be credibly statistically significant without support from prior evidence.

## References

[1] Jeffreys H. 1939 Theory of probability, pp. 388–389. Oxford, UK: Oxford University Press.

[2] Yates F 1951 The influence of statistical methods for research workers on the development of the science of statistics. JASA 46, 19–34.

[3] Edwards W, Lindman H, Savage LJ 1963 Bayesian statistical inference for psychological research. Psychol. Rev. 70, 193–242. (doi:10.1037/h0044139)

[4] Open Science Collaboration. 2015 Estimating the reproducibility of psychological science. Science 349, 943 (doi:10.1126/science.aac4716) 10.1126/science.aac4716 26315443

[5] Baker M 2016 1,500 scientists lift the lid on reproducibility. Nature 533, 452–454. (doi:10.1038/533452a) 27225100 10.1038/533452a

[6] Wasserstein RL, Lazar NA 2016 The ASA's statement on *p*-values: context, process, and purpose. Am. Stat. 70, 129–133. (doi:10.1080/00031305.2016.1154108)

[7] Matthews RAJ, Wasserstein R, Spiegelhalter D 2017 The ASA's *p*-value statement, one year on. Significance 14, 38–41. (doi:10.1111/j.1740-9713.2017.01021.x)

[8] Matthews RAJ 2001 Why should clinicians care about Bayesian methods? J. Stat. Inf. Plan. 94, 43–58. See also discussion, 59–71 (doi:10.1016/S0378-3758(00)00232-9)

[9] Matthews RAJ 2001 Methods for assessing the credibility of clinical trial outcomes. Drug. Inf. J. 35, 1469–1478. (doi:10.1177/009286150103500442)

[10] Spiegelhalter DJ 2004 Incorporating Bayesian ideas into health-care evaluation. Stat. Sci. 19, 156–174. (doi:10.1214/088342304000000080)

[11] Rothman KJ, Greenland S, Lash TL (eds). 2008 Modern epidemiology, Ch 18 Philadephia, PA: Lippincott Williams & Wilkins.

[12] Greenland S 2011 Null misinterpretation in statistical testing and its impact on health risk assessment. Prev. Med. 53, 225–228. (doi:10.1016/j.ypmed.2011.08.010) 21871481 10.1016/j.ypmed.2011.08.010

[13] Held L 2013 Reverse-Bayes analysis of two common misinterpretations of significance tests. Clin. Trials. 10, 236–242. (doi:10.1177/1740774512468807) 23329516 10.1177/1740774512468807

[14] Berkson J 1942 Tests of significance considered as evidence. JASA. 37, 325–335. (doi:10.1080/01621459.1942.10501760) 10.1093/ije/dyg255 14559729

[15] Goodman S 2008 A dirty dozen: twelve *p*-value misconceptions. Semin. Hematol. 45, 135–140. (doi:10.1053/j.seminhematol.2008.04.003) 18582619 10.1053/j.seminhematol.2008.04.003

[16] Ziliak ST 2016 The significance of the ASA Statement on Statistical Significance and *p*-values. Commentary to [6]. (doi:10.1080/00031305.2016.1154108)

[17] Fisher RA. 1925 Statistical methods for research workers. Edinburgh, UK: Oliver & Boyd.

[18] Rothman KJ 1978 A show of confidence. NEJM 299, 1362–1363. (doi:10.1056/NEJM197812142992410) 362205 10.1056/NEJM197812142992410

[19] Gardner MJ, Altman DG 1986 Confidence intervals rather than *p* values: estimation rather than hypothesis testing. BMJ 292, 746–750. (doi:10.1136/bmj.292.6522.746) 3082422 10.1136/bmj.292.6522.746 PMC1339793

[20] Lee PM 1997 Bayesian statistics: an introduction. London, UK: Arnold.

[21] Belia S, Fidler F, Williams J, Cumming G 2005 Researchers misunderstand confidence intervals and standard error bars. Psych. Meth. 10, 389–396. (doi:10.1037/1082-989X.10.4.389) 10.1037/1082-989X.10.4.389 16392994

[22] McGrayne SB 2011 The theory that would not die. New Haven, CT: Yale University Press.

[23] Good IJ 1950 Probability and the weighing of evidence, Ch. 4, pp. 35–36 London, UK: Griffin.

[24] Spiegelhalter DJ, Abrams KR, Myles JP 2004 Bayesian approaches to clinical trials and health-care evaluation, Ch. 3 Chichester, UK: Wiley & Sons.

[25] Taubes G, Mann CC 1995 Epidemiology faces its limits. Science 269, 164–169. (doi:10.1126/science.7618077) 7618077 10.1126/science.7618077

[26] Kabat GC 2016 Getting risk right: understanding the science of elusive health risks. New York, NY: Columbia University Press.

[27] Goodman SN 2016 The next questions: who, what, when, where, and why? Online commentary to Wasserstein & Lazar [6]. (doi:10.1080/00031305.2016.1154108)

[28] Hankins MC 2013 Still not significant. Probable Error blog entry. See https://mchankins.wordpress.com/2013/04/21/still-not-significant-2/.

[29] Altman DG, Bland JM 1995 Statistics notes: absence of evidence is not evidence of absence. BMJ 311, 485 (doi:10.1136/bmj.311.7003.485) 7647644 10.1136/bmj.311.7003.485 PMC2550545

[30] Greenland S, Senn SJ, Rothman KJ, Carlin JB, Poole C, Goodman SN, Altman DG 2016 Statistical tests, *p* values, confidence intervals and power: a guide to misinterpretations. Euro. J. Epidem. 31, 337–350. (doi:10.1007/s10654-016-0149-3) 10.1007/s10654-016-0149-3 PMC4877414 27209009

[31] Berry DA 2016 *P*-values are not what they're cracked up to be. Online commentary to [6]. (doi:10.1080/00031305.2016.1154108)

[32] Benjamin DJ et al. 2017 Redefine statistical significance. Nat. Hum. Behav. 1 (doi:10.1038/s41562-017-0189-z) 10.1038/s41562-017-0189-z 30980045

[33] Colquhoun D 2014 An investigation of the false discovery rate and the misinterpretation of *p*-values. R. Soc. open sci. 1, 140216 (doi:10.1098/rsos.140216) 26064558 10.1098/rsos.140216 PMC4448847

[34] Colquhoun D 2017 The reproducibility of research and the misinterpretation of *p*-values. R. Soc. open sci. 4, 171085 (doi:10.1098/rsos.171085) 29308247 10.1098/rsos.171085 PMC5750014

[35] Johnson VE 2017 Revised standards for statistical evidence. Proc. Natl Acad. Sci. USA. 110, 19 313–19 31 7. (doi:10.1073/pnas.1313476110) 10.1073/pnas.1313476110 PMC3845140 24218581

[36] Gelman A, Carlin J 2017 Some natural solutions to the *p*-value communication problem – and why they won't work. JASA 112, 899–901. (doi:10.1080/01621459.2017.1311263)

